# Einsatz eines virtuellen Kollaborators in analogen & digitalen Workshops im organisationalen Kontext

**DOI:** 10.1007/s00287-021-01361-z

**Published:** 2021-05-06

**Authors:** Nicole Debowski, Navid Tavanapour, Eva A. C. Bittner

**Affiliations:** Fakultät für Mathematik, Informatik und Naturwissenschaften Informatik, Universität Hamburg, Vogt-Kölln-Straße 30, 22527 Hamburg, Deutschland

## Abstract

In dieser Studie stellen wir Unterschiede und Gemeinsamkeiten in der analogen sowie digitalen Zusammenarbeit hinsichtlich eines virtuellen Kollaborators (VK) dar. Konkret beobachten wir eine Kreativeinheit in einem Industrieunternehmen sowohl in der analogen als auch in der digitalen Durchführung kollaborativer Workshops. Aus den daraus resultierenden Herausforderungen und Anforderungen, die wir anhand von Interviews erheben, leiten wir Designprinzipien an einen VK ab und ziehen einen Vergleich. Gemeinsamkeiten bestehen darin, den Teilnehmenden zusätzliche Informationen und kreativen Input aus internetbasierten Quellen zu liefern. Unterschiede bestehen in der administrativen Vor- und Nachbereitung der jeweiligen Workshops sowie in der Art der Beeinflussung kollaborativer Arbeit. Während bei der digitalen Durchführung eher die Perspektiverweiterung im Vordergrund steht, ist es bei der analogen Durchführung die Ausbalancierung der Redebeiträge. Spezifika stellen sich darüber hinaus für die digitale Durchführung bei der Vernetzung der Teilnehmenden sowie beim Umgang mit digitalen Werkzeugen.

## Einleitung

Aufgrund der Covid-19-Pandemie und der seit März 2020 wiederkehrenden Lockdown-Situationen ist die Arbeitswelt gezwungen, innerhalb kürzester Zeit von einer präsenzorientierten und analogen zu einer vollständig virtuellen Umgebung überzugehen. Beschäftigte sind daher mit virtuellen Werkzeugen konfrontiert worden, mithilfe derer sie zur Erfüllung ihrer beruflichen Tätigkeiten zusammenarbeiten [1]. Ein Szenario für eine solche Zusammenarbeit sind digitale Workshops innerhalb von Organisationen, die vor der Pandemiesituation vor Ort durchgeführt wurden. Neben Vorteilen bringt virtuelle Zusammenarbeit auch neue Herausforderungen für Moderierende und Teilnehmende der Workshops mit sich. Im virtuellen Setting fehlt es etwa an nonverbaler Kommunikation und Interaktion, was zu einer anderen Teamatmosphäre führen kann. Darüber hinaus werden Moderierende und Teilnehmende mit mehreren verschiedenen Kommunikations- und Informationsströmen gleichzeitig über Sprache und Text konfrontiert. Auch vermeintlich kleine Interaktionen wie Zeigen, Hervorheben oder Organisieren erfordern stets eine Werkzeugfunktion und virtuellen Aufwand, was bei Workshops vor Ort weniger kompliziert ist. Diese können zu einer Überlastung führen und erfordern eine automatisierte Unterstützung von virtuellen Workshops.

Eine solche Unterstützung kann mit einem virtuellen Kollaborator (VK) gestaltet werden. Dieser basiert auf einem technologiebasierten Agenten, der in der Lage ist, seine Umgebung wahrzunehmen, Informationen zu verarbeiten, Entscheidungen zu treffen und zu lernen, auf sie einzuwirken und mit Menschen und anderen Maschinen zu interagieren, um ein gemeinsames Aufgabenziel mit mehr oder weniger Autonomie zu erreichen [2]. Beispiele dafür sind Sprachassistenten wie Siri von Apple, Alexa von Amazon oder Cortana von Microsoft. Aufgrund ihrer konstanten und schnellen Entwicklung sowie ihrer Anpassbarkeit an die Bedürfnisse der Menschen wird die Zusammenarbeit mit technologiebasierten Agenten immer attraktiver [3]. Ein VK geht dahingehend jedoch weiter: Er ist nicht nur auf Assistenzfunktionen beschränkt, sondern als ein gleichberechtigter virtueller Teamkollege in einer kollaborativen Arbeitsumgebung zu betrachten, der mit dem Benutzer agiert [4].

Nass & Moon (2000) zeigten etwa in ihrer Social-Response-Theorie, dass Nutzer von Computern diese durchaus menschlich behandeln, etwa indem sie ihnen menschliche soziale Kategorien wie Geschlecht oder Ethnie zuteilen, höflich gegenüber dem Computer sind oder den Computer als „Spezialisten“ bezeichnen [5]. Langer (1992) bezeichnete dieses Verhalten als „mindless behavior“, als unbedachtes Verhalten, bei dem Individuen auf zuvor erlernte Konzepte und Muster zurückgreifen, ohne sich des Gegenübers bewusst zu werden [6, 7].

Eine große Anzahl von Forschungsartikeln befasst sich bereits mit der Zusammenarbeit zwischen Mensch und Technologien wie künstlicher Intelligenz (KI), und zeigt ein großes Potenzial der KI für die Zukunft der Arbeit auf [8–12]. Spezifische Faktoren wie Vertrauen und Skepsis gegenüber der KI [13, 14] werden bereits erforscht. Es mangelt jedoch an einem ganzheitlichen Ansatz und besonders an Gestaltungswissen für KI-gestützte virtuelle Zusammenarbeit im Kreativitätsprozess. Insbesondere die organisationale Sichtweise ist noch nicht ausreichend erforscht, was bereits von Forschenden über Forschungsagenden [15, 16] und Podiumsdiskussionen [2] eingefordert wird.

An dieser Stelle positionieren wir unsere Forschung und untersuchen aus organisationaler Sicht, welchen Herausforderungen Moderierende und Teilnehmende in kreativen digitalen Workshops im Vergleich zu analogen Workshops gegenüberstehen und wie ein VK gestaltet werden kann, um den identifizierten Herausforderungen zu begegnen. Zu diesem Zweck stellen wir 2 Forschungsfragen auf.

*F1: Wie unterscheiden sich die Designprinzipien eines VK für digitale Workshopsituationen von denen für analoge Workshopsituationen?*

*F2: Welche Designprinzipien lassen sich aus organisationaler Perspektive für einen VK explizit für digitale Kreativworkshops definieren?*

Diese Forschungsfragen zielen auf Designprinzipien (DP) für einen VK in digitalen Workshopsituationen ab. Durch einen Vergleich zwischen DPs für digitale Workshopsituationen und denen für analoge werden spezifische Besonderheiten in der Gestaltung für virtuelle Settings herausgearbeitet. In diesem Zusammenhang wurde eine 2‑phasige Interviewstudie innerhalb einer Kreativeinheit eines Industrieunternehmens (KEI) durchgeführt, die im Nachfolgenden genauer erläutert wird. In einer ersten Iteration wurden analog durchgeführte Workshops mit verschiedenen Fachbereichen wie Logistik oder IT betrachtet, die in diesem Artikel als Referenz für die Veränderungsbedarfe dienen. Die zweite Iteration beleuchtet digital durchgeführte Workshops genauer. Im vorliegenden Artikel werden die Herausforderungen, Anforderungen und Designprinzipien in analog und digital durchgeführten Kreativworkshops miteinander verglichen.

Wir verfolgen einen explorativen qualitativen Ansatz, indem wir semistrukturierte Interviews mit Teilnehmenden und Moderierenden von Kreativitätsworkshops durchführten, die von einer KEI durchgeführt wurden. Zunächst wird die Forschungsumgebung – die digitale und analoge Durchführung von Workshops der KEI – an einem Beispiel vorgestellt. Anschließend präsentieren wir unsere Ergebnisse mit den identifizierten Anforderungen (AF) sowie den Designprinzipien bei analogen (aDP) und digitalen (dDP) Workshops für den VK. Wir setzen die Diskussion unserer Ergebnisse in Verbindung mit vorhandener Literatur fort und skizzieren die Grenzen unserer Forschung. Zum Schluss ziehen wir ein Fazit und stellen unseren Beitrag heraus.

## Wissenschaftliche Vorgehensweise

### Kreativeinheiten in Industrieunternehmen als Forschungsumgebung

**Figure 1:**
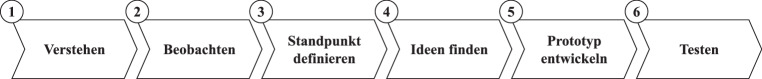

Im Folgenden wird zunächst die Arbeitsweise der untersuchten KEI im analogen sowie digitalen Kontext erläutert. Die KEI orientiert ihre Arbeit stark am Design-Thinking-Ansatz im 6‑Phasen-Modell nach Meinel et al. (2009) (siehe Abb. 1; [17]). Design Thinking (DT) ist eine Methode zur Lösung bestehender Probleme, und bindet frühzeitig verschiedene Stakeholder mit unterschiedlichem Hintergrund ein [18]. Im Vordergrund stehen dabei konsequent die Bedürfnisse der Nutzer, die in jeder Phase einbezogen werden. DT folgt einem strukturierten, iterativen Prozess, in dem ein multidisziplinäres Team verschiedene (Kreativitäts‑)Methoden zur Erreichung des Phasenziels einsetzt [19].

Je nach Phase im DT-Prozess wird ein thematisch passender Workshop konzipiert, was im Folgenden beispielhaft sowohl in der analogen als auch in der digitalen Durchführung anhand der dritten Phase „Standpunkt definieren“ erläutert wird. Schallmo & Lang (2020) beschreiben diese Phase wie folgt: „Die [aus den vorherigen Phasen] gewonnenen Erkenntnisse werden ausgewertet, interpretiert und gewichtet. Dabei werden die Erfahrungen des gesamten Teams zusammengefasst, um eine gemeinsame Basis zu schaffen. Hierbei wird eine typische, fiktive Person (‚Persona‘) erstellt, die ganzheitlich beschrieben wird. Dabei ist es wichtig, relevante Fakten von nicht relevanten Fakten zu trennen.“ [20].

Durchgeführt wird der analoge Workshop in einem Raum mit Utensilien zum kreativen Arbeiten wie Whiteboards, Stifte, verschiedene Arten von Papier und anderes Zubehör zum Visualisieren und Gestalten [21]. Zu Beginn des Workshops wird durch die Moderierenden eine Einleitung sowie ggf. eine Vorstellung der Teilnehmenden gegeben, sofern sich diese noch nicht kennen. Dann wird die Agenda vorgestellt. Teil der Agenda sollten nicht nur inhaltliche und praktische Bestandteile sein, sondern auch Pausen [22, 23].

Danach beginnt die inhaltliche Arbeit. Ziel der hier beschriebenen „Standpunkt-definieren“-Phase ist es, die zuvor geführten Interviews zu sichten, zu synthetisieren und zu analysieren. Dabei werden die Interviewinhalte auf Klebezettel geschrieben und auf dem Whiteboard verteilt. Anschließend spricht das Team über die Inhalte: Welche Erkenntnisse gab es? Was war überraschend? Was wurde besonders häufig genannt? Auf was wurde gar nicht eingegangen? Solche und weitere Fragen können bei der Synthese der Interviewinhalte hilfreich sein. Ist das Team mit den Inhalten vertraut, können verschiedene Visualisierungstechniken eingesetzt werden [24].

Anschließend werden die gewonnenen Informationen, z. B. in einer User Journey, zusammengefasst. Die User Journey stellt den Weg der Nutzung einer Lösung dar. Dabei sollen vor allem die unterschiedlichen Phasen und Bedürfnisse in diesen sowie die Erfahrungen und Kontaktpunkte der Nutzer dargestellt werden [25]. Dafür wird ein Template mit den entsprechenden Feldern erstellt, das unter Nutzung eines Whiteboards befüllt wird. Die Teammitglieder besprechen gemeinsam die Inhalte für die entsprechenden Felder und füllen diese per Hand aus. Die Moderation als neutrale Stelle achtet während des gesamten Prozesses vornehmlich darauf, die Diskussionen zu unterstützen, etwa durch Fragen oder eigene Impulse. Nach Durchsprache aller Einzelaufgaben werden die nächsten Schritte diskutiert. Dabei kann es vorkommen, dass aufgrund der iterativen Natur des DT-Prozesses das Team einen Schritt zurückgeht, etwa merkt, dass noch weitere Informationen zu den potenziellen Nutzerinnen und Nutzern notwendig sind [21]. Ist der Workshop beendet, muss das Moderationsteam den Workshop nacharbeiten und eine lückenlose Dokumentation des Workshops anfertigen.

Digitale Workshops erfolgen bei der KEI ebenfalls nach dem oben beschriebenen Prozess sowie den entsprechenden Prinzipien im DT, unterscheiden sich jedoch in der Durchführung. Während bei analogen Workshops ein physischer Raum zur Verfügung gestellt wird, finden digitale Workshops im virtuellen Raum statt. Die hier vorgestellte KEI nutzt Microsoft Teams als Kollaborationstool, vornehmlich die Konferenzfunktion. Vorbereitet wird der Workshop durch entsprechende Erläuterungen und Templates mithilfe von Powerpoint-Folien, die in der Konferenz präsentiert werden. Konkret wird zunächst jeder einzelne Schritt mündlich und zusätzlich schriftlich auf der Folie erläutert. Im nächsten Schritt werden die Teilnehmenden in Gruppen von maximal 6 Personen aufgeteilt. Jeder Gruppe steht ein Moderationsteam bestehend aus 2 Personen zur Verfügung. Die Gruppen können mithilfe der „Break-out-rooms“-Funktion in Microsoft Teams automatisch randomisiert gebildet werden [26].

Dann startet die inhaltliche Arbeitsphase mit den vorbereiteten Templates. Dabei stehen dem Moderationsteam 2 Varianten zur Verfügung: Entweder schreiben die Teilnehmenden direkt in die Templates, etwa beim lauten Brainstorming [27, 28]. Oder aber die Teilnehmenden schreiben ihre Gedanken zunächst mithilfe der Chatfunktion von Microsoft Teams für sich selbst auf [29]. Ist die Bearbeitungszeit abgelaufen, gibt das Moderationsteam ein Signal, bei dem alle Teilnehmenden gleichzeitig ihre niedergeschriebenen Gedanken in den Gruppenchat senden. Anschließend präsentiert jedes Teammitglied und das Moderationsteam überträgt die vorgestellten Inhalte zu Dokumentationszwecken vom Gruppenchat in das vorbereitete Template. Dies eignet sich besonders, wenn Teilnehmende noch nicht häufig mit dem verwendeten Programm gearbeitet haben. Nach diesem Prinzip werden alle Arbeitsphasen durchgeführt. Auch hier wird abschließend über nächste Schritte gesprochen und eine Feedbackrunde durchgeführt. Durch die digitale Bearbeitung in den Templates während des Workshops entfällt die Nachbereitung zumeist oder kann dadurch zumindest stark eingegrenzt werden.

## Durchführung qualitativer Interviews und ihre Resultate

Wir erhoben Daten aus der Perspektive potenzieller Nutzer eines VK, indem wir 2 Interviewreihen mit jeweils 12 halbstrukturierten Interviews mit einer Dauer von 30–40 min durchführten [30]. In den Interviews wurden zunächst Herausforderungen analoger und digitaler Workshops sowie Anforderungen an einen VK, bezogen auf diese, behandelt. Anschließend wurden Unterschiede zwischen der analogen und der digitalen Durchführung sowie den entsprechenden Anforderungen an einen VK für diese diskutiert.

Die Interviewpartner wurden nach ihrer Rolle als Moderierende (M) oder Teilnehmende (T) sowie nach der jeweiligen Workshoperfahrung ausgewählt, die zuvor als Kriterium definiert wurden (Tab. 1 und 2). Die Workshoperfahrung wurde für die Moderierenden anhand der Anzahl der durchgeführten digitalen Workshopmoderationen (Hoch > 50; Durchschnittlich < 50 Workshops) seit der Lockdown-Situation durch die Covid-19-Pandemie bemessen. Die Workshoperfahrung für Teilnehmende wurde anhand der Anzahl der teilgenommenen digitalen Workshops (Hoch > 10 Workshops; Durchschnittlich < 10 Workshops) seit der Lockdown-Situation durch die Covid-19-Pandemie bemessen.

**Table 1:** 

Interviewte	Workshoperfahrung	Relevante Ausbildung
aM1-aM3	Hoch	Zertifizierter DT Expert
aM4	Hoch	Agile Coach
aM5	Durchschnittlich	Keine, Praxiserfahrung
aT1	Durchschnittlich	In Ausbildung zum DT Expert
aT2	Durchschnittlich	Keine
aT3-aT7	Durchschnittlich	Keine

**Table 2:** 

Interviewte	Workshoperfahrung	Relevante Ausbildung
dT1-dT2	Hoch	Häufig teilgenommen
dT3-dT5	Durchschnittlich	Häufiger teilgenommen
dM1-dM3	Durchschnittlich	Keine, Praxiserfahrung
dM4-dM5	Hoch	In Ausbildung zum DT Expert
dM6-dM7	Hoch	Zertifizierter DT Expert

## Resultate und Diskussion

### Vergleich zwischen analog und digital

**Figure 2:**
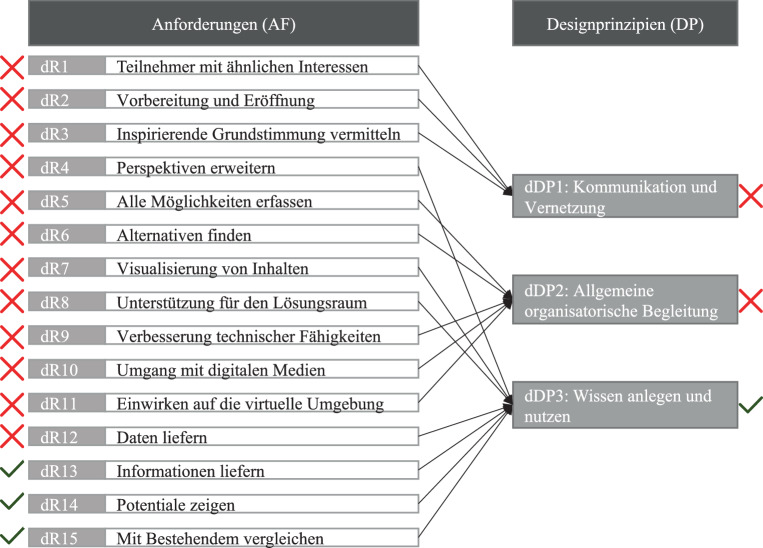

Im Folgenden wird auf *F1* eingegangen, und die Unterschiede und Gemeinsamkeiten zwischen der analogen und digitalen Workshopdurchführung explizit dargestellt. Abb. 2 zeigt Designprinzipien für einen VK für digitale Workshops mit Übereinstimmungen (grüner Haken) und Abweichungen (rotes Kreuz).

#### Unterschiede

Der größte und am häufigsten genannte Unterschied von virtuellen zu analogen Workshops ist das Fehlen von Körpersprache und direktem Feedback. Die Chance, einen ersten Eindruck von einer Situation und ihren Teilnehmenden zu bekommen, fällt vollständig weg. Oft ist die Atmosphäre zu Beginn eines Workshops sehr zurückhaltend und die Teilnehmenden sprechen nicht miteinander, bis das Moderationsteam den Workshop offiziell eröffnet hat. Während es in digitalen Workshops durch die mangelnde Körpersprache also eher problematisch ist, die Teilnehmenden zu vernetzen und anzuregen, zu sprechen und sich zu beteiligen, ist es in analogen Workshops eher problematisch, die Gruppe ausbalanciert sprechen zu lassen und ein gemeinsames Ziel zu verfolgen. Aus diesem Grund wird die Objektivität des VKs vorausgesetzt, um eine neutrale Instanz innerhalb der Gruppe zu schaffen und um sicherzustellen, dass der Input von jedem Teilnehmenden gleich behandelt wird *(aAF1, aDP1)*: „Nicht 100 % neutral, aber ich denke, die Wahrnehmung wäre, dass sie zumindest auf der Basis der Daten, die sie aufnimmt, alles gleich behandelt und nicht etwas anderes als untergeordnet“ – aT10. In zahlreichen Studien wurde bereits untersucht, dass KI-gestützte Systeme die Rolle eines empathischen Teammitglieds übernehmen können, das Feedback zum Team gibt und auch kritische Punkte anspricht [2, 31–34].

Die administrative Vorbereitung eines analogen Workshops wie die Organisation und Gestaltung wurde häufig als Herausforderung genannt. Das Protokollieren und das Aufzeigen von Zusammenhängen zwischen verschiedenen Ergebnissen wurden als sehr zeitaufwendig beschrieben, ebenso das Erstellen von Protokollen und das Ausfüllen von Vorlagen [35]. Dies wird auch vom VK erwartet: „Ich würde jetzt erwarten, dass ich ein Transkript des Gesprächs bekomme und der Mitarbeiter die Nachbereitung oder so macht“ – aM2 *(aAF3, aDP1)*. Eine Studie von Dolata et al. (2019) etwa zeigte, dass KI-fähige Systeme in der Lage sind, bei administrativen Arbeiten wie dem Erfassen, Transkribieren und Archivieren von Dokumenten und Sitzungsprotokollen während des Workshops zu helfen oder über verschiedene Wege wie E‑Mails oder Messenger zu kommunizieren [36]. Bei der digitalen Durchführung hingegen war eine dezidierte Planung und Vorbereitung unabdingbar, und erforderte mehr Zeit und Aufwand als bei der analogen Variante. Es war notwendig, alle Möglichkeiten, die während des Workshops auftreten könnten, durchzugehen und gleichzeitig Alternativen vorzubereiten, falls etwas nicht klappen sollte *(dAF5, dAF6, dDP2)*. Darüber hinaus mussten die Ziele des Workshops genauer definiert werden, um die Methoden und den Prozess darauf abzustimmen: „Man muss den ganzen Workshop öfter durchgehen, was sind die Möglichkeiten in den interaktiven Sitzungen, und welche Alternativen haben wir“ (dM4). Durch die Nutzung digitaler Medien wird die Nachbereitung wiederum wesentlich erleichtert. Hier zeigen zahlreiche Studien, dass ein VK bei der Entscheidungsfindung helfen kann, indem dieser verschiedene Denkschritte bei einer komplexen Entscheidung repräsentiert [37, 38].

aM1, aM2 und aM4 erwähnten, dass es mitunter schwierig sein kann, geeignete Methoden zu wählen, in andere Methoden zu wechseln oder aber auch neue Methoden auszuprobieren. Dahingehend könnte der VK Methoden vorschlagen, die auf dem Workshopziel *(aAF4, aDP3)* oder der Stimmung der Teilnehmenden *(aAF5, aDP1)* basieren, um besser auf die Bedürfnisse der Gruppe einzugehen: „Auch Methoden, wie man so ein Thema angeht, damit halt die durchführende Person einfach eine Zielsetzung hat, was sie eigentlich machen soll“ – aT7. Es wurde in mehreren Studien festgestellt, dass KI-fähige Systeme in der Lage sind, Gruppenentscheidungsprozesse sowie Teamergebnisse zu beeinflussen, um die Ziele ihrer Nutzer zu erreichen [32, 39, 40].

Es wurde häufig genannt, dass in der analogen Durchführung eher der Fokus darauf lag, auf die Teilnehmenden einzugehen, während die Problematik bei der digitalen Durchführung eher darin bestand, den Lösungsraum im DT allgemein kreativ anzugehen: „Die ersten drei Phasen des Design-Thinking-Prozesses sind digital gut machbar, danach wird es schwieriger“ (dM7). Dies liegt daran, dass die letzten 3 Phasen mehr Visualisierungsmöglichkeiten benötigen *(dAF7, dDP3)*, die in der digitalen Umsetzung eher schwierig sind. Daher ist die klare Notwendigkeit, die Ergebnisse durch den VK über (bewegte) Icons, Symbole oder auch Wortwolken visuell zu unterstützen und aufzubereiten (dM4). Cautela et al. (2019) untersuchten in einer Studie, inwieweit derzeit die DT-Praxis mithilfe von KI übernommen oder unterstützt werden kann. Während die ersten 3 Phasen des DT-Prozesses – wie etwa Recherchen, Vorschläge geben und Daten analysieren – gut umzusetzen sind, fehlt es an Applikationen, die auf die Ideenfindung und andere kreative Prozesse eingehen [41].

#### Gemeinsamkeiten

Neben diesen Unterschieden gab es jedoch auch einige Gemeinsamkeiten in der digitalen und analogen Durchführung von Kreativworkshops. Hinsichtlich analoger Workshops wurde erwähnt, dass die eigene Meinung während einer Workshopsituation zurückgehalten wurde, weil man der Meinung war, dass es an entsprechendem Wissen oder Erfahrung fehlte, um wertvolle Beiträge zu leisten: „Ich spreche erst, wenn ich von meinen Gedanken halbwegs überzeugt bin“ – aT6. Um dieser Herausforderung vorzubeugen bzw. entgegenzuwirken, wird vom VK erwartet, dass er schnell und zuverlässig *(aAF6, aDP2) *Informationen sammelt und verarbeitet, wie Studien von Waizenegger (2020) und Seeber (2019) zeigen [14, 31]. Auch in digitalen Workshops soll der VK den Prozess durch die Bereitstellung von Informationen und Hintergrundwissen unterstützen (dT2). Durch semantische Analysen und Stichwortsuchen sollen nicht nur Daten *(dAF12, dDP3)*, sondern auch breites Wissen *(dAF13, dDP3)* zur Unterstützung der Recherchephasen generiert werden: „Der VK könnte breites Wissen zu bestimmten Stichworten in visualisierter Form anbieten“ – dT2. Weitere Studien zeigen, dass von KI-fähigen Systemen erwartet wird, dass sie Muster sammeln, analysieren, synthetisieren und identifizieren, die in den vorgestellten Workshopsituationen direkt genutzt werden können [42, 43].

Für digitale Workshops wurde häufig angeführt, dass der VK in der Lage sein sollte, die Idee eines Anwenders im Hinblick auf Potenziale und Einsatzmöglichkeiten *(dAF14, dDP3)* sowie auf bereits bestehende oder ähnliche Ideen *(dAF15, dDP3)* durch eine Verbindung zum Internet zu analysieren (dT2). Für analoge Workshops sollte diese Fähigkeit genutzt werden, um bestehende Lösungen, etwa aus sozialen Medien *(aAF7, aDP2)*, als weiteren Impuls oder Anregung zu finden: „Es kann einen Hinweis geben, indem man auf soziale Medien oder andere Quellen verweist und sich davon inspirieren lässt“ – Ma3. Studien zeigen, dass von KI-fähigen Systemen erwartet wird, dass sie Muster sammeln, analysieren, synthetisieren und identifizieren, die in den vorgestellten Workshopsituationen direkt genutzt werden können [42, 43].

Das Sprechen vor dominanteren Teilnehmenden war in beiden Fällen problematisch, da nicht nur einzelne Teilnehmende, sondern die gesamte Gruppe von der Meinung der dominanten Person beeinflusst wird, was zu einer einseitigen Workshopstruktur führen kann. Aus diesem Grund wird vom VK erwartet, dass er die Redeanteile überwacht *(aAF2, aDP1)*: „Dass jeder über ein Gadget verbunden ist, dass er dann merkt ‚ok hier sind 2 oder 3 Leute nicht so aktiv im Gespräch‘“ – aT7. Damit sollen die Redebeiträge gemessen und entweder der gesamten Gruppe oder nur dem Moderierenden transparent gemacht werden, um notwendige Maßnahmen zur Wiederherstellung der Balance zu treffen.

Auch in digitalen Workshops war eine ausbalancierte Diskussion gewünscht, jedoch in Form einer Perspektivenerweiterung *(dAF4, dDP3)*, z. B. als Sparringspartner zum Austausch von Meinungen (dM2, dT2). Da KI-gestützte Systeme verschiedene Aufgaben übernehmen, aber nicht von einem Menschen geleitet und kontrolliert werden, können so traditionelle Macht- und Kontrollhierarchien infrage gestellt werden. Dies kann einen direkten Einfluss sowohl auf die Ergebnisse eines Workshops als auch auf das Verhalten der dominanteren Teilnehmenden haben [44].

## Spezifische Designprinzipien für digitale Workshops

Im folgenden Abschnitt wird auf *F2* eingegangen und spezifische DPs für digitale Workshops entwickelt. Aus den Herausforderungen und Anforderungen wurden nach dem Design-Science-Research-Ansatz von Hevner et al. (2000) und Gregor et al. (2020) DPs abgeleitet [45, 46].

### Kommunikation und Vernetzung

Um Teilnehmende mit ähnlichen Interessen und Fähigkeiten zusammenzubringen *(dAF1, dDP1)*, sollte der VK in der Lage sein, Informationen aus Datenbanken und Netzwerken aus dem Intra- und Internet zu sammeln und zusammenzubringen (dM2). Dies sollte ein Anreiz zur Vernetzung und zum Austausch sein. Im Vorfeld könnte auch ein workshopspezifisches Profil erstellt werden. Die Vernetzung sollte jedoch nicht zu aktiv, sondern eher unterschwellig und subtil erfolgen: „So etwas wie ‚Kennen Sie XY? Sie ist auch agiler Coach‘, basierend auf dem, was in meinem LinkedIn- oder Intranet-Profil steht“ – dM2. Weiterhin sollte der VK als Gastgeber verschiedene virtuelle Räume für unterschiedliche Situationen zur Verfügung stellen und mit einem Begrüßungstext und der Einleitung einer Vorstellungsrunde *(dAF2, dDP1)* ansprechend gestalten (dT1, dM4): „Der VK könnte durch die Räume gehen, er könnte sagen: ‚Hier ist, was auf der Tagesordnung steht‘, wie ein Chatbot“ (dT1).

Der VK könnte hinsichtlich der fehlenden Körpersprache und mangelnden Vernetzung der Teilnehmenden unterstützen, indem dieser anhand von Stimm- und Gesprächsanalysen Stimmungen erkennt und entsprechende Tipps an die Moderierenden gibt oder direkt steuernd auf die Teilnehmenden einwirken (dT1, dM6). Gleichzeitig könnte auch eine öffnende und inspirierende Grundhaltung vermittelt werden *(dAF3, dDP1)*, um den kreativen Prozess in den Workshops anzuregen: „Wenn die Leute offen sind, eine Art Coaching durch den VK zu bekommen, könnte das helfen“ (dM6). Tseng et al. (2019) zeigen, dass ein VK auch bei der Bildung von Teams helfen kann, indem die Mitgliederkandidaten auf psychologische Weise analysiert werden, um passende Profile zu identifizieren [45]. Daneben zeigen Kocielnik et al. (2018) und Tseng et al. (2019), dass ein VK auch bei Reflektionsphasen unterstützen kann, was wiederum Coachingaktivitäten von Moderierenden unterstützen kann [47, 48].

### Allgemeine organisatorische Begleitung

Ein weiterer wichtiger Faktor ist die Arbeit mit digitalen Werkzeugen. Sowohl Moderierende als auch Teilnehmende nannten technische Fähigkeiten und den Umgang mit digitalen Medien als wichtige Faktoren für eine erfolgreiche digitale Durchführung *(dAF9, dAF10, dDP2)*. Der VK sollte in der Lage sein, Fragen über Text zu beantworten oder unbeantwortete Fragen an die Moderierenden am Ende des Workshops weiterzugeben (dT1, dM5, dM6). In Form eines Chatbot soll der VK Tipps und Tricks im Umgang mit virtuellen Schnittstellen aufzeigen, um die Kompetenzen der Teilnehmenden im Umgang mit den Medien zu erweitern (dT1, dM5, dM6). Außerdem sollte der VK die Teilnehmenden und Moderierenden während des gesamten Kollaborations- und Kreativitätsworkshops begleiten (dT1). Dabei sollte der VK organisatorisch und unterstützend auf die unmittelbare virtuelle Umgebung einwirken *(dAF11, dDP2)* (dT1). Ein VK wird in der Literatur darüber hinaus ohne anthropomorphen Hintergrund vorgeschlagen, z. B. zum Zugriff auf Gebäudeinformationen [49] oder zur Verbesserung der Zugänglichkeit von Unternehmensinformationen [50]. Dies könnte entsprechend auf Informationen zum Umgang mit Medien übertragen werden.

## Fazit, Beitrag und Grenzen

In dieser Studie stellen wir Unterschiede und Gemeinsamkeiten in der analogen sowie digitalen Zusammenarbeit mit einem VK dar. Hinsichtlich *F1 *zeigen wir einerseits deutliche Unterschiede in den DPs auf. In digitalen Workshops liegt der Schwerpunkt auf der Vernetzung, der kreativen Arbeit sowie auf dem hohen Aufwand bei der Vorbereitung. Demgegenüber steht bei analogen Workshops eher im Fokus, eine ausbalancierte Diskussion zu erreichen, Einfluss auf die Gruppe insgesamt zu nehmen sowie den Aufwand in der Nachbereitung zu reduzieren. Gemeinsamkeiten bestehen in der Wissenserzeugung und der Informationsbereitstellung. Hinsichtlich *F2 *zeigen wir DPs auf, die spezifisch für einen VK in digitalen Workshops gelten. So soll ein VK in digitalen Workshops die fehlende Körpersprache durch Anreize zum Vernetzen der Teilnehmenden sowie zur Kommunikation miteinander überwinden, und Unterstützung im Umgang mit digitalen Werkzeugen und einer virtuellen Arbeitsumgebung bieten.

Besonders im Fokus steht in der Beantwortung von *F1* und *F2* der praktische Nutzen unserer Studien. Wir haben im vorliegenden Vergleich insbesondere die Unterstützung eines VK im organisationalen Kontext einer Kreativeinheit untersucht. Wir bringen dabei spezifische DPs im Hinblick auf ein soziotechnisches System ein, die als Grundlage für die weitere Forschung im Bereich der virtuellen Zusammenarbeit und der Unterstützung der Zusammenarbeit in einem organisatorischen Umfeld dienen kann, das zuvor meist an einem gemeinsamen Standort arbeitete [2, 31, 51]. Wir spezifizieren Herausforderungen, die sich aus der digitalen Zusammenarbeit von Teilnehmenden ergeben, die von zu Hause aus arbeiten und versuchen, Lehren aus der physischen Zusammenarbeit in die digitale Durchführung von Kreativitätsworkshops zu übernehmen, dargestellt als Anforderungen.

Unsere Studien weisen einige Grenzen auf. Wir haben bisher nur eine KEI in nur einer Industrie betrachtet. Weitere Forschung könnte einen branchenübergreifenden Blick auf die Thematik geben. Dazu gehören auch unterschiedliche Herangehensweisen in den Kreativeinheiten. Zudem wurden in diesen Studien analoge und digitale Workshops getrennt voneinander betrachtet. In zukünftiger Forschung kann eine Verknüpfung von analogen Workshops mit digitaler Unterstützung diskutiert werden. Außerdem kann der Fokus verstärkt auf den DT-Prozess gelegt werden, indem untersucht wird, an welcher Stelle im DT-Prozess ein VK eine Entlastung für Moderierende darstellen kann. Aufbauend auf unseren Studien eignet sich abschließend eine empirische Untersuchung, um die qualitativ entwickelten Inhalte zu validieren.
